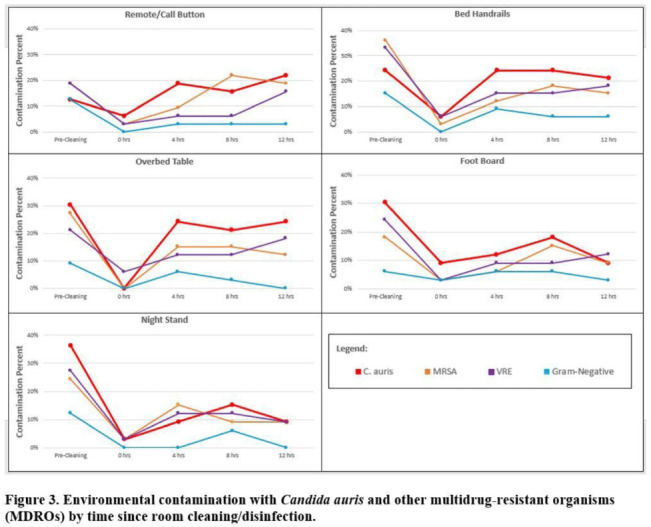# Multicenter evaluation of contamination of the healthcare environment near patients with *Candida auris* skin colonization

**DOI:** 10.1017/ash.2022.205

**Published:** 2022-05-16

**Authors:** Sarah E. Sansom, Gabrielle M. Gussin, Raveena D Singh, Pamela B Bell, Ellen Benson, Jinal Makhija, Mary Carl Froilan, Raheeb Saavedra, Robert Pedroza, Christine Thotapalli, Christine Fukuda, Ellen Gough, Stefania Marron, Maria Del Mar Villanueva Guzman, Julie A. Shimabukuro, Lydia Mikhail, Stephanie Black, Massimo Pacilli, Hira Adil, Cassiana E. Bittencourt, Matthew Zahn, Nicholas Moore, D.J. Sexton, Judith Noble-Wang, Meghan Lyman, Michael Lin, Susan Huang, Mary K. Hayden

## Abstract

**Background:** *Candida auris* is an emerging multidrug-resistant yeast that is transmitted in healthcare facilities and is associated with substantial morbidity and mortality. Environmental contamination is suspected to play an important role in transmission but additional information is needed to inform environmental cleaning recommendations to prevent spread. **Methods:** We conducted a multiregional (Chicago, IL; Irvine, CA) prospective study of environmental contamination associated with *C. auris* colonization of patients and residents of 4 long-term care facilities and 1 acute-care hospital. Participants were identified by screening or clinical cultures. Samples were collected from participants’ body sites (eg, nares, axillae, inguinal creases, palms and fingertips, and perianal skin) and their environment before room cleaning. Daily room cleaning and disinfection by facility environmental service workers was followed by targeted cleaning of high-touch surfaces by research staff using hydrogen peroxide wipes (see EPA-approved product for *C. auris*, List P). Samples were collected immediately after cleaning from high-touch surfaces and repeated at 4-hour intervals up to 12 hours. A pilot phase (n = 12 patients) was conducted to identify the value of testing specific high-touch surfaces to assess environmental contamination. High-yield surfaces were included in the full evaluation phase (n = 20 patients) (Fig. 1). Samples were submitted for semiquantitative culture of *C. auris* and other multidrug-resistant organisms (MDROs) including methicillin-resistant *Staphylococcus aureus* (MRSA), vancomycin-resistant *Enterococcus* (VRE), extended-spectrum β-lactamase–producing Enterobacterales (ESBLs), and carbapenem-resistant Enterobacterales (CRE). Times to room surface contamination with *C. auris* and other MDROs after effective cleaning were analyzed. **Results:** *Candida auris* colonization was most frequently detected in the nares (72%) and palms and fingertips (72%). Cocolonization of body sites with other MDROs was common (Fig. 2). Surfaces located close to the patient were commonly recontaminated with *C. auris* by 4 hours after cleaning, including the overbed table (24%), bed handrail (24%), and TV remote or call button (19%). Environmental cocontamination was more common with resistant gram-positive organisms (MRSA and, VRE) than resistant gram-negative organisms (Fig. 3). *C. auris* was rarely detected on surfaces located outside a patient’s room (1 of 120 swabs; <1%). **Conclusions:** Environmental surfaces near *C. auris*–colonized patients were rapidly recontaminated after cleaning and disinfection. Cocolonization of skin and environment with other MDROs was common, with resistant gram-positive organisms predominating over gram-negative organisms on environmental surfaces. Limitations include lack of organism sequencing or typing to confirm environmental contamination was from the room resident. Rapid recontamination of environmental surfaces after manual cleaning and disinfection suggests that alternate mitigation strategies should be evaluated.

**Funding:** None

**Disclosures:** None

**Figure:**
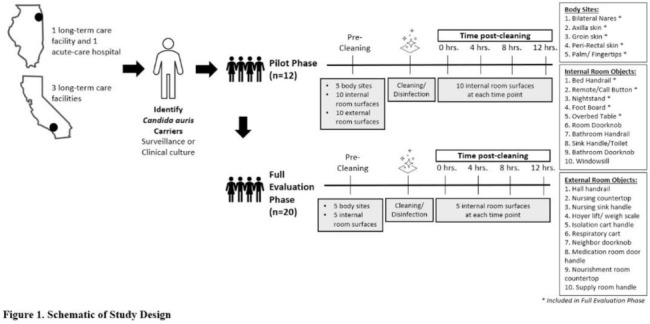

**Figure:**
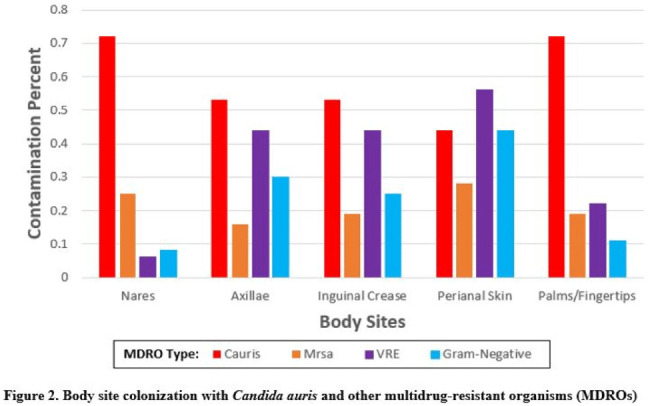

**Figure:**