# Multicenter evaluation of contamination of the healthcare environment near patients with Candida auris skin colonization – ERRATUM

**DOI:** 10.1017/ash.2022.306

**Published:** 2022-10-07

**Authors:** Sarah E. Sansom, Gabrielle M. Gussin, Raveena D Singh, Pamela B Bell, Ellen Benson, Jinal Makhija, Mary Carl Froilan, Raheeb Saavedra, Robert Pedroza, Christine Thotapalli, Christine Fukuda, Ellen Gough, Stefania Marron, Maria Del Mar Villanueva Guzman, Julie A. Shimabukuro, Lydia Mikhail, Stephanie Black, Massimo Pacilli, Hira Adil, Cassiana E. Bittencourt, Matthew Zahn, Nicholas Moore, D. Sexton, Judith Noble-Wang, Meghan Lyman, Michael Lin, Susan Huang, Mary K. Hayden

In the above article^1^, the following author changes have been made:

1. Author Raheen Froilan has been corrected to “Mary Carl Froilan”.
2. Sarah Sansom's name has been corrected to “Sarah E. Sansom”.
3. Author Ellen Benson Jinal has been corrected to “Ellen Benson”.
4. Author Makhija has been corrected to “Jinal Makhija”.
5. Author Mary Hayden has been corrected to “Mary K. Hayden”.
